# The radius of influence of a combined method of in situ air sparging and soil vapor extraction in the intertidal sediments of Gomso Bay on the west coast of South Korea

**DOI:** 10.1186/s40064-016-3026-3

**Published:** 2016-08-22

**Authors:** Jun-Ho Lee, Han Jun Woo, Kap-Sik Jeong, Kap-Song Park

**Affiliations:** 1 Korean Seas Geosystem Research Center, Korea Institute of Ocean Science & Technology (KIOST), 787, Haean-ro, Sangnok-gu, Ansan-si, Gyeonggi-do 15627 Korea; 2 Department of Environmental Science and Engineering, Hankuk University of Foreign Studies (HUFS), 81, Oedae-ro, Mohyeon-myeon, Cheoin-gu, Yongin-si, Gyeonggi-do 17035 Korea

**Keywords:** Effective oxygen, In situ air sparging, Radius of influence, Soil vapor extraction, Tidal flat

## Abstract

**Background:**

In situ air sparging (IAS) was undertaken at sites in the tidal flats of Mandol and Hajeon, on the west coast of South Korea, to estimate variations in the radius of influence (ROI).

**Results:**

The Mandol core sample consisted of sand and muddy sand 1.6–3.4 $\Phi$ (average 2.3 $\Phi$) and contained water (average 15.10 %). The Hajeon core sample consisted of muddy sand, sandy silt, and muddy sandy gravel 1.31–4.44 $\Phi$ (average 3.11 $\Phi$) and contained water (average 19.77 %). These sites differ in their sedimentary and geochemical characteristics. At the Mandol site, no H_2_S or combustible gas was detected during a 48-h sampling period, except for some volatile organic compounds (0.1–2.0 ppm) at the monitoring well during the initial 30 min. At the soil vapor extraction wells, CO_2_ and O_2_ varied by 850 ppm (690–1540 ppm) and 0.5 % (20.4–20.9 %), respectively. At the Hajeon site, CO_2_ and O_2_ varied from 580 to 1250 ppm and 20.6 to 20.9 %, respectively, during the 48-h sampling period.

**Conclusions:**

At the Mandol site, an oxygen concentration of 20.6 % was assumed as the effective concentration, and the ROI was estimated to be 128.0 cm. However, at the Hajeon site the ROI was estimated to be 85.7 cm. The smaller effective ROI at the Hajeon site was likely caused by the thin aquifer and thin screens of the sparing well. This estimated ROI show that the remediation effectiveness varies greatly as a heterogeneities and anisotropies in the porous sediments. Besides, injection pressure, flow rate, pulsing or continuous mode, and the range of intrinsic permeability for most important characteristic of sediment (soil) type impacted the ROI. Therefore, the IAS method is more effective at a pervasive air flow sediments such as Mandol, which consists of sand and muddy sand than at a channelized site such as Hajeon.

**Electronic supplementary material:**

The online version of this article (doi:10.1186/s40064-016-3026-3) contains supplementary material, which is available to authorized users.

## Background

In situ air sparging (IAS) has been used since the mid-1980s, mainly as an inland underground water remediation technique to remove polluted oil, the uncontrolled disposal of wastewater, and industrial discharges. The technique treats or settles sediments and dissolved volatile organic compounds (VOCs) by injecting air into the saturated zone. Compared to other techniques, IAS technologies require smaller facilities and relatively low energy inputs. Pump and treatment processes in specific hydro-geologic settings must be carefully reviewed before air sparging (AS) can be used to remediate sites contaminated by VOCs (Ji et al. 1993; Nyer and Suthersan 1993). A soil vapor extraction (SVE) method has been developed for hydrophobic pollutants, which do not dissolve easily in water. This technology is effective, especially in the vadose zone. Bioremediation technology is being further developed for a wide variety of such purposes. It is used in combination with other purifying technologies to upgrade the remediation efficiency in polluted soils or underground aquifers (Chao et al. 2008). Also many petrochemical products are likewise produced to be almost sulfur-free (Javadli and Klerk 2012). The Monte Carlo (MC) analysis was conducted using the mean intrinsic permeability value of 1.2 × 10^−11^ m^2^ indicated from descriptive available field observations (Mallette et al. 2012; Rahbeh and Mohtar 2007).

Although AS was introduced in 1985 (Chao et al. 2008; Shevah and Waldman 1995), laboratory experiments at the time failed to clearly reveal the relationship between particle size and airflow, as well as the area of influence and the pattern of the saturated air within natural sediments (Peterson et al. 2000, 1999). Fully understanding the pervasive airflow, which occurs during groundwater pollution remediation using IAS, is essential (Abdel-Moghny et al. 2012; Koretsky et al. 2006).

Further in situ experiments (Lundegard and Labrecque 1995) identified two parameters, radius of influence (ROI) in the saturated zone (ROI_SAT_) and in the vadose zone (ROI_VAD_) that provided information about the size of the area influenced by airflow (Ghabayen et al. 2013). Characteristics of the porous medium have significant effects on contaminant removal and remediation for system design factors. The IAS, airflow distributions are directly impacted by air permeability, particle size, and grain sizes (Benner et al. 2002). The ROI is determined by the grain sizes and air channels formed by injected air (Mohtar et al. 1996). And an evaluation using model simulations (BIOVENTING^PLUS^) suggested that IAS operation for pulsed air injection or continuous air flow were significant in terms of removal of a critical species, total xylenes (Benner et al. 2000).

Pilot tests are an important method to upgrade full scale for understanding of IAS behavior remediation site. But prediction of long-time performance based on pilot tests has proved to be difficult. Before to pilot test activities, it is important to evaluate the anticipated operating pressure for the IAS system (Johnson et al. 1997). Minimum injection pressure is the general procedure for the minimum required to initiate sparging and maximum pressures which should be applied to the aquifer layer sediments.

The number of injection and extraction wells installed to purify a contaminated site generally depends on the distribution and depth of contamination. For optimal remediation of contaminated sites, the ROI of the drill hole should be clearly identified and further, the number and positions of drill holes required for the remediation of corresponding contaminated areas should be accurately counted to avoid the passing over of contaminated areas. Additionally, care should also be taken to prevent potential excessive investment owing to overlapped or duplicated design of required facilities (Fan et al. 2013; Chai and Miura 2004).

This study was undertaken at two sites in Gomso Bay, on the west coast of Korea, where various types of natural sediment are distributed under the influence of the tide. By assuming a scenario in which the bay was contaminated by an oil slick, this study aimed to estimate the applicability of IAS techniques on a pilot scale to the physical, chemical, and biological remediation of coastal sediments together with a determination of the site-specific elements that influence the efficiency of the technique.

## Methods

### Sparging well design

**Fig. 1:**
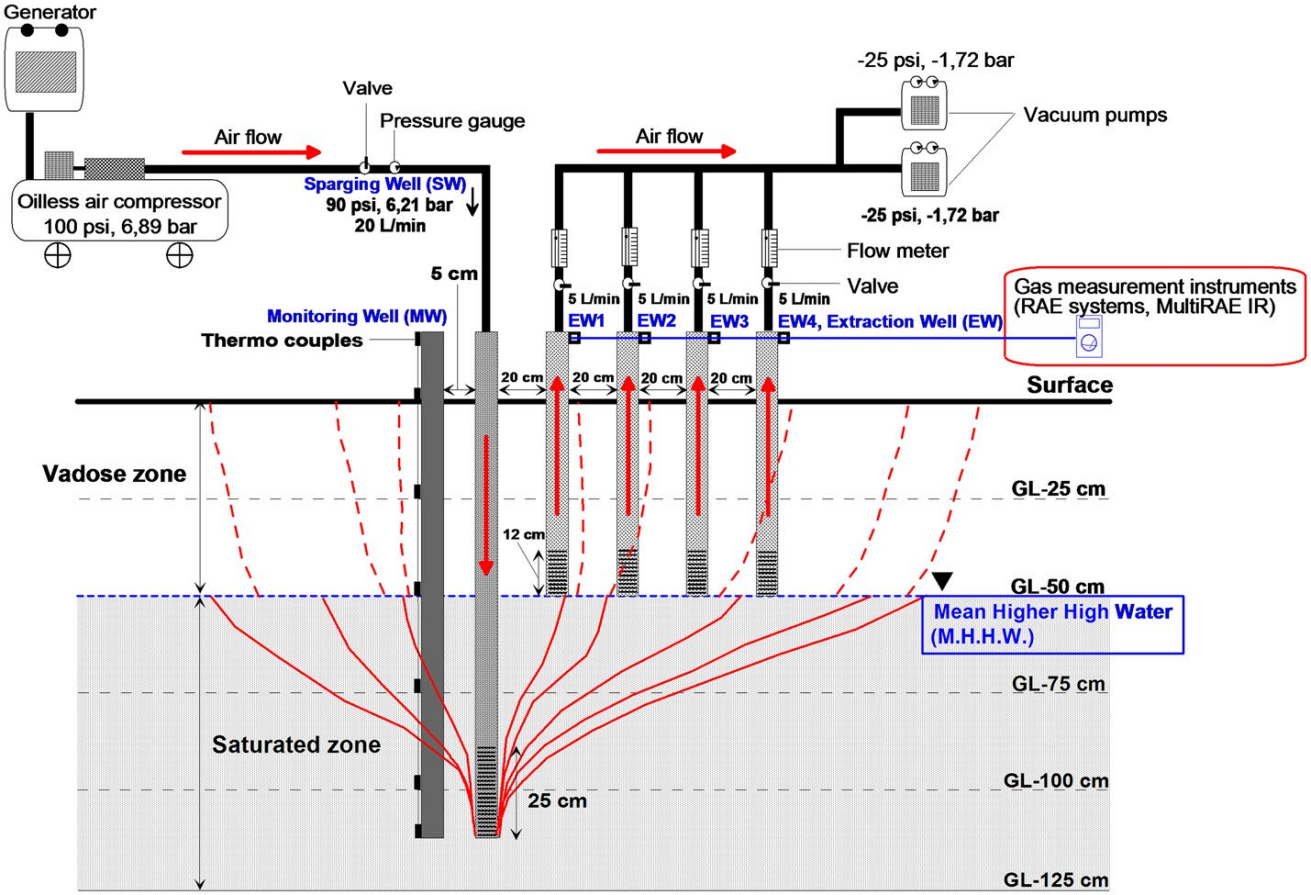
Schematic diagram of the monitoring, sparging, and vapor extraction wells

At each site, the experimental setup consisted of one monitoring well (MW), one sparging well, and four SVE wells (Fig. 1). The MW was 120 cm deep, with a series of seven thermocouples installed at regular intervals. The sparging well was 110 cm deep, with a screen (25 cm depth) placed at the bottom to disseminate the air that was pumped in. The screen contained a series of 2 mm wide air holes at 1 cm intervals. The SVE well was 60 cm deep with a screen (12 cm depth) also placed at the bottom. The MW was established 5 cm from the sparging well.

The sparging well and SVE wells were established 20 cm apart. To prevent seawater from entering through the SVE well screen during floods, the SVE wells were located at the mean higher high water (MHHW) level. The air injected through the sparging well was regulated to be released at a rate of 20 L/min (90 psi (6.21 bar), minimum injection pressure) into the saturated zone, and the injected air was regulated to pass through SVE wells 1–4 at a rate of 5 L/min (–25 psi) each. To inject air into the sparging well, an oil-free air pressure pump was used. The concentration of CO_2_ and O_2_ was measured at arbitrary time intervals of 0, 3, 6, 27, 31, and 48 h. The air pressure throughout the sparging well was 90 psi (6.21 bar, minimum injection pressure) during the 48 h when air was injected. Air from the SVE wells 1–4 was exhausted using two vacuum pumps (−25 psi, −1.72 bar ×2). The concentrations of H_2_S, VOCs, CO_2_, lower explosion level (LEL), and O_2_ were periodically measured by a MultiRAE IR^®^ (RAE Systems Inc., San Jose, CA, USA) connected to the MW and SVE wells 1–4.

### Sampling and analysis of core sediments

Following AS or extraction, core sediments were taken within 5 m of the site where air was injected by pushing and sampling methods (Carter and Gregorich 2006). To retrieve the sediments, a transparent acrylic pipe (8 cm diameter) was extended to depths of 25–70 cm. The top and bottom of the sediment core were sealed with styrofoam and stoppers. The acrylic pipes were cut vertically with a diamond-coated round saw, while taking care not to mix the sediments. Sediments were then cut into halves with a guitar string, after the color, biological materials, sedimentary structures, and particle sizes were determined. Subsamples of the sediments were collected from five depths beginning at 5 cm from the bottom of the core.

At regular intervals in the cores, particle size, water content (WC), total carbon (TC), total organic carbon (TOC), total nitrogen (TN), and bulk density (BD) were measured, and a soft X-radiograph was taken after the shear strength was measured using a fall cone. A 1 × 30 cm vertical slab was taken from the core and photographed using X-ray apparatus (Softex Co., Ebina-City, Kanagawa, Japan).

For particle size analysis, core sediment samples of about 5 g were placed in a 1-L beaker and treated with 10 % hydrogen peroxide (H_2_O_2_) to completely remove organics. The removal of carbonates was achieved by adding 0.1 N hydrochloric acid (HCl) to the solution. Each sample was separated into finer and coarser fractions using a 4 $\Phi$ (62 µm) sieve. The coarser fractions were sieved for 15 min using a Ro-Tap sieve shaker, with sieves mounted at 0.5 $\Phi$ intervals, and weight percentages were obtained for each particle size class. Samples of the finer fraction were placed in a diffusing solution (0.1 % Calgon) and evenly dispersed with an ultrasonicator and a magnetic vibrator prior to analysis with an X-ray automatic particle size analyzer (Sedigraph 5100, Micromeritics Instruments Corp., Norcross, GA, USA). Statistical variables such as the mean, sorting, skewness, and kurtosis were obtained for the weight percentages calculated for each particle size class according to a moment method (McManus 1998; Folk 1968; Krumbein 1934).

For the analysis of TOC, 1 N HCl (10 mL) was added overnight to powdered sediment samples (0.5 g) to remove carbonates. Samples were then reweighed, and the difference in weight was calculated. A few milligrams of experimental material was placed in an aluminum tin capsule and TC, TOC, and TN were measured using an elementary analyzer (EA1112, Thermo Electron Co., Waltham, MA, USA). To ensure the reliability of the analytic data, a soil reference material (SRM), a standard reference material of the National Institute of Standards and Technology (NIST) of the United States, was also analyzed and compared to the results obtained.

The WC may be expressed by weight as the ratio of the mass of moisture present to the dry weight of the sediment samples. To determine these ratios for sediment samples, the water mass was determined by drying the soil to constant weight and measuring the mass of the soil sample before and after drying. The soil sample was dried to constant weight in an oven at 105 °C. The moisture content on a dry weight basis was calculated using the following Eq. (1):

1 $$\theta_{d} \,(water\,content) = \frac{{\left( {water\,content\,of\,wet\,sediment} \right)\, - \,(water\,content\,of\,dry\,sediment)}}{(water\,content\,of\,dry\,sediment)}$$

BD was determined using a pycnometer (Accupyc 1330, Micromeritics Instruments Corp.) and water rate values were adjusted. Each sample from Mandol and Hajeon was analyzed three times, and function rate values were adjusted to find an average bulk density value.

To determine the shear strength using a fall cone apparatus, 60 g (Mandol) or 10 g (Hajeon) of sediment at constant penetration of a cone was directly proportional to the weight of the cone, and the relationship between the shear strength and the penetration of a cone of weight *Q* is given by Eq. (2):

2 $$s = k\frac{Q}{{h^{2} }},$$

where *k* is a constant that depends mainly on the angle of the cone, but is also influenced by the sensitivity of the clay. The relationship between the depth of penetration and undrained shear strength is given in the enclosed tables. The standard cone used in these measurements had a capacity of 60 g and an angle of 60°.

### Minimum injection pressure

Outlined are the equations for the general procedure for estimating the minimum pressure required to initiate sparging and the maximum pressure that should be exerted on the aquifer (Reddy and Adams 1996). The operating pressure for an IAS system will be determined by the depth of the IAS and the permeability of the aquifer. The minimum injection pressure necessary to induce flow (P_min_ (psi,gas)) is given by Eq. (3):

3 $$P_{min} \left( {psi, gas} \right) = Factor1 \cdot (0.43 \cdot H_{h} + P_{packing} + P_{pormation} ),$$

The pressure at which fracturing of the aquifer can occur is given by Eq. (4):

4 $$P_{fraction} \left( {psi, gas} \right) = Factor2 \cdot (0.73 \cdot D),$$

where *H*_*h*_ = depth from the water table to the screened section of the injection well (the hydrostatic head) (ft); *P*_*packing*_ and *P*_*pormation*_ = air entry pressures for the well packing material and the formation (psig); *Factor*1 and *Factor*2 = sparging coefficients in the field test; and *D* = depth from the ground surface to the screened section of the air injection well (ft). For typical IAS wells and applications, *P*_*packing*_ and *P*_*pormation*_ are small compared to the contribution from the hydrostatic head (air entry pressures are generally <0.2 psig for sands, <0.4 psig for silts, but may be >1.5 psig in some clay rich settings). At start-up, it is not unusual to exceed *P*_*min*_ by as much as 5–10 psig to initiate flow quickly. The injection pressure then generally declines to about *P*_*min*_ as steady flow conditions are approached. Pressures in excess of *P*_*fraction*_ can cause fracturing of the formation; however, as the pressure drops off rapidly when moving away from an injection point, the extent of fracturing, in most cases, is expected to be limited to the area immediately surrounding the well. In this experiment, the 90 psi (6.21 bar, minimum injection pressure, “Sparging well design” section) air injection to the sparging well can be summarized as Eqs. (5) and (6). The values of Factor1 and Factor2 in the sparging coefficient were 81.8 and 37.7 in the field test, respectively.

$$P_{min} \left( {psi, gas} \right) = Factor1 \cdot 0.43 \cdot H_{h} + P_{packing} + P_{pormation}$$

5 $$P_{min} \left( {psi, gas} \right) = 89.98,$$

$$Factor1 = 81.8, \,H_{h} = 1.64\, {\rm ft}, \,P_{packing} = 0.2 {\rm psi} \,and\, P_{pormation} = 0.2\,{\rm psi}$$

and

$$P_{fraction} \left( {psi, gas} \right) = Factor2 \cdot 0.73 \cdot D$$

6 $$P_{fraction} \left( {psi, gas} \right) = 90.10,$$

$$Factor2 = 37.7\, and \, D = 3.28 ft$$

## Study area

**Fig. 2:**
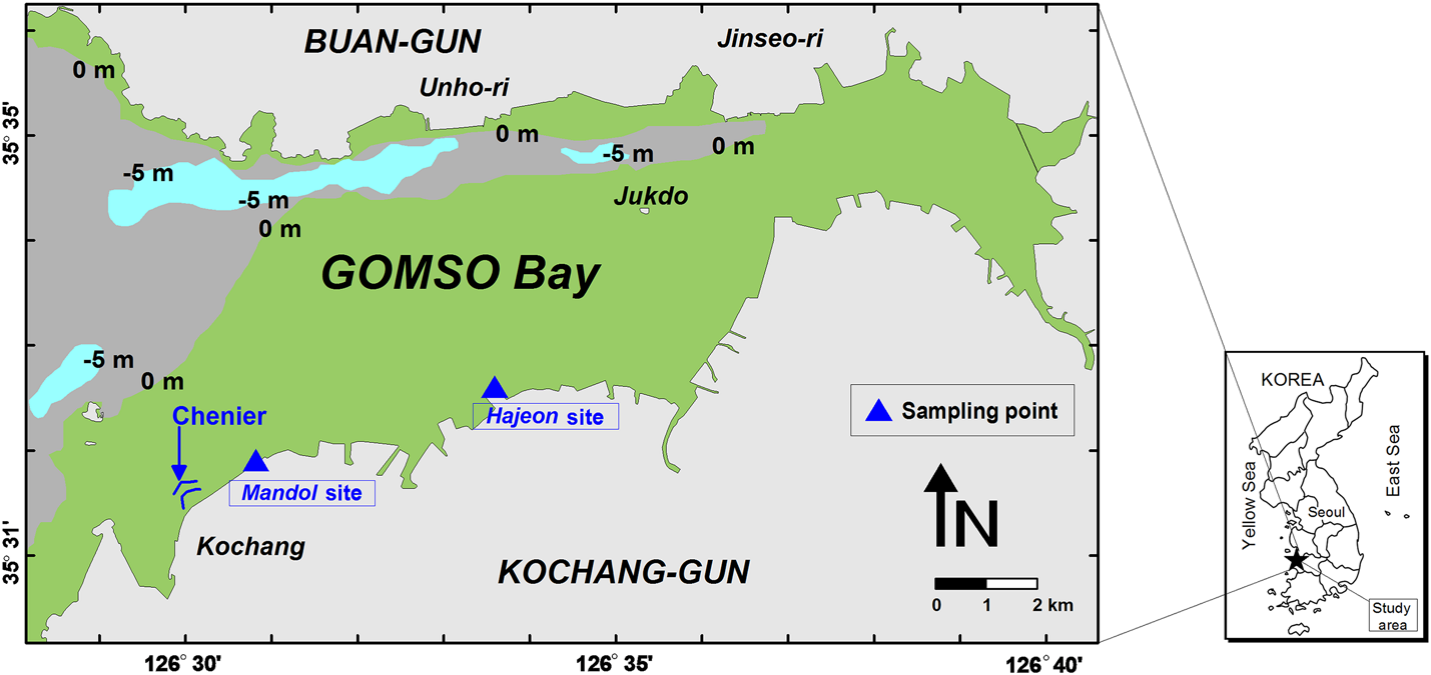
Location of IAS experiments combined with SVE

The construction of a small embankment along the shoreline has impacted the tidal and coastal currents in Gomso Bay by changing the topography of the mud flat. However, the bay still remains in good natural condition and is largely free from pollution due to the low population and limited number of industrial facilities in the surrounding area (Chang et al. 2007). A small river (Jujeonchun River) flows through the southern middle part of the tidal flat and provides an influx of freshwater (Google map 2016). The tidal flat contains well-developed sub-environments such as the foreshore, tidal route, tidal creek, and chenior. The IAS experiment was undertaken at two sites (Mandol and Hajeon) in the upper intertidal zone (Table 1; Fig. 2; Additional files: 1, 2, 3, 4, 5, 6).

**Table 1:** IAS experimental sites in Gomso Bay on the west coast of Korea

Station		DM		Core sediment type
		Longitude (E)	Latitude (N)	
Gomso Bay	Mandol	126° 30.7512′	35° 31.8552′	Sand, muddy sand
	Hajeon	126° 33.5932′	35° 32.5900′	Muddy sand, silty sand, sandy silt, sandy silt, muddy sandy gravel

The tide changes twice a day in Gomso Bay with almost no diurnal inequality. The mean range is 433.8 cm (spring: 589.8 cm, neap: 277.8 cm) and the maximum range reaches 717.4 cm (Chang et al. 2007). The semidiurnal flood current velocity is 115 cm/s in Gomso Bay, and the ebb current is 150 cm/s; thus, the ebb flow tends to be more dominant (Ryang and Shon 2003). The intertidal zone is made up of sand (S), silty sand (zS), sandy silt (sZ), and silt (Z) that become finer toward the coast. This is probably a result of changes to the hydrodynamic conditions resulting from human activities (Chang et al. 2007).

**Fig. 3:**
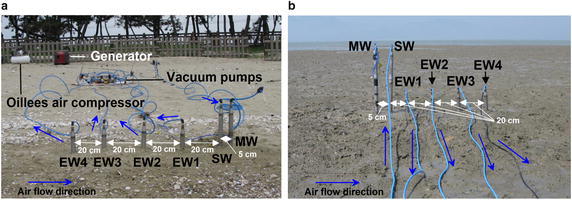
Photograph of an IAS experiment combined with SVE. **a** Mandol site. **b** Hajeon site

During the experiment at Mandol, the air temperature was 16.5–24.7 °C (KMA 2012). The average wind direction (measured every 10 min) was largely SSE on both May 31 and June 1, with an average wind speed of 1.0–4.9 m/s, mean atmospheric pressure of 1009.22 hPa, and cloud cover of 6.3–9.8 %. Continuous air injection by IAS and extraction through SVE occurred for 48 h from 15:00 on May 31 to 15:00 on June 1 (local time). The initial temperature at the MW was recorded as 21.0–27.0 °C (measured for 1 min), the radiation intensity of the atmosphere was 350–380 × 100 Lux (measured for 1 min), and atmospheric moisture was 48.1 % (measured for 1 min at 25.5 °C; Fig. 3a; Additional file 7).

At Hajeon, air injection by IAS and extraction through SVE occurred for 48 h from 15:00 on June 1 to 15:00 on June 3 (local time). The initial temperature at the MW was 21.0–27.0 °C for (measured for 1 min), the radiation intensity of the atmosphere was 350–380 × 100 Lux (measured for 1 min), and atmospheric moisture was 48.1 % (measured for 1 min at 25.5 °C; Fig. 3b). It was cloudy with a relatively low temperature during the test in Mandol while the weather was fine during the test in Hajeon.

## Results

### Textural and geochemical characteristics of core sediments

**Fig. 4:**
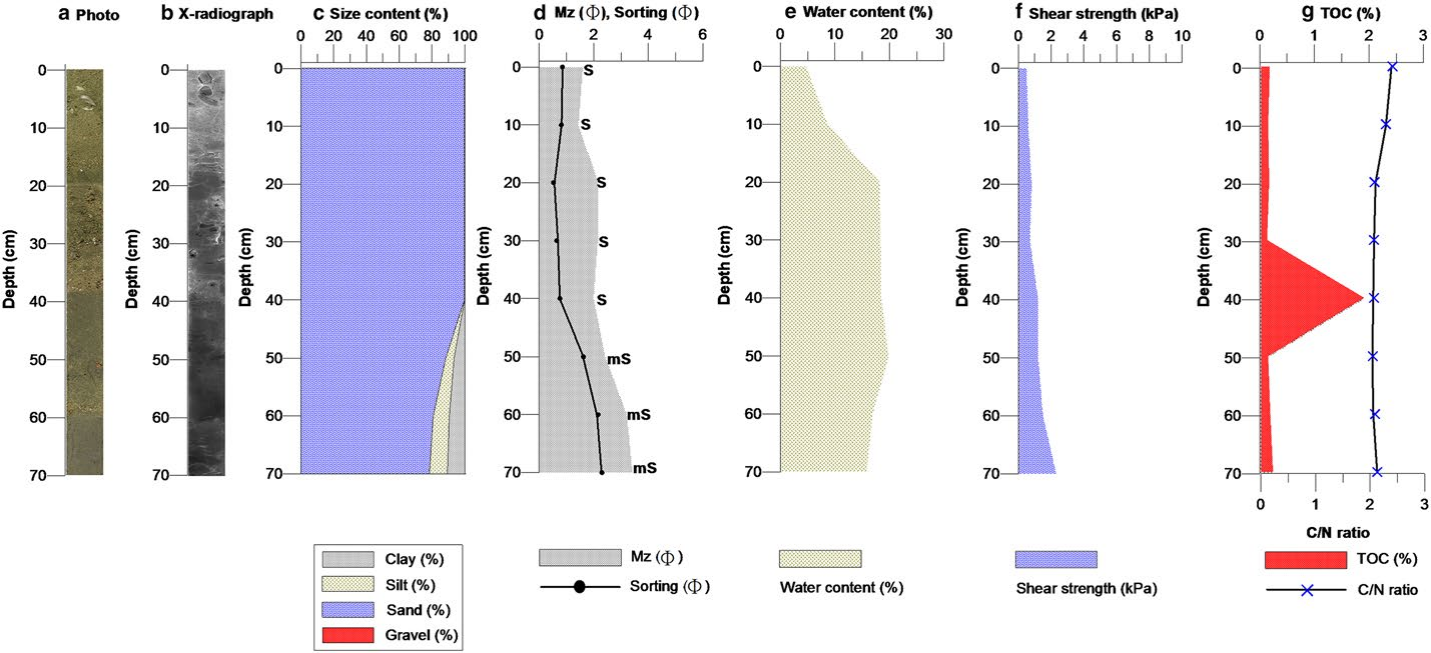
**a** Cross-sectional photographs showing, **b** a soft X-radiograph, **c** textural composition, **d** mean grain size and sorting, **e** water content, **f** shear strength, and **g** total organic carbon and C/N ratio in the sediment core from Mandol

The core taken from the Mandol site contained an abundance of shell fragments, which were generally an olive color (5Y/6) using soil color (Munsell 2000), from the surface to a depth of 45 cm. These were also detected on the soft X-radiograph (Fig. 4). The proportion of sand was greater than 80 % to a depth of 60 cm: sand and muddy sand [Mz 1.60–3.41 $\Phi$ (average 2.30 $\Phi$), sorting 0.52–2.30 $\Phi$ (average 1.21 $\Phi$), and WC 4.69–19.85 % (average 15.10 %)]. The shear strength was 0.5–2.3 kPa (average 1.1 kPa) in the top part of the core, which consisted mostly of sand. TOC and TN varied with depth. TOC had a range of 0.11–1.89 % (average 0.37 %) and TN had a range of 0.00–0.12 % (average 0.02 %). A large proportion of the sand in the core had low organic carbon content (Tables 2, 3; Fig. 4; Additional file 8). The Mandol core, with a higher proportion of sand, was lower in organic carbon content.

**Table 2:** Textural composition (%), mean-grain size ($\Phi$), sediment type, sorting ($\Phi$), water content (%), and shear strength (kPa) of core sediments from Mandol and Hajeon

Depth (cm)	Gravel (%)	Sand (%)	Silt (%)	Clay (%)	Mean-grain size ($\Phi$)	Sediment type by Folk	Sorting ($\Phi$)	Water content (%)	Shear strength (kPa)
Mandol									
0	0.00	99.83	0.17	0.00	1.60	S	0.85	4.69	0.5
10	0.00	99.78	0.22	0.00	1.44	S	0.81	8.66	0.6
20	0.00	99.89	0.11	0.00	2.16	S	0.52	18.20	0.8
30	0.00	99.78	0.22	0.00	2.15	S	0.64	18.34	0.7
40	0.00	99.9	0.10	0.00	1.99	S	0.76	18.43	1.2
50	0.00	88.36	5.15	6.49	2.42	mS	1.62	19.85	1.2
60	0.00	80.78	9.81	9.41	3.21	mS	2.16	16.88	1.5
70	0.00	78.45	11.0	10.55	3.41	mS	2.30	15.79	2.3
Ave.	0.00	93.35	3.35	3.31	2.30	–	1.21	15.11	1.10
Hajeon									
0	0.00	53.89	26.45	19.66	4.44	mS	3.49	18.84	1.0
5	0.00	60.69	28.07	11.24	3.32	zS	2.90	18.51	0.9
10	0.00	45.77	37.59	16.64	4.62	sZ	3.39	19.93	1.5
15	0.00	62.67	23.02	14.31	3.60	mS	3.18	23.47	1.8
20	30.04	48.14	11.83	9.99	1.36	msG	3.90	19.98	1.8
25	36.92	35.03	15.91	12.14	1.31	msG	4.51	17.86	3.0
Ave.	11.16	51.03	23.81	14.00	3.11	–	3.56	19.77	1.67

Folk, sediment types (after Folk 1968)

*S* sand, *mS* muddy sand, *zS* sility sand, *sZ* sandy silt, *msG* muddy sandy gravel; (g) *sM* slightly gravelly muddy sand, *gM* gravelly mud, *mG* muddy gravel

**Table 3:** Total carbon (TC), total organic carbon (TOC), total nitrogen (TN), sulfur (S), and bulk density (BD) of core sediments from Mandol and Hajeon

Depth (cm)	TC	TOC	TN	S	Bulk density
	(%)	(%)	(%)	(%)	(g/cm^3^)
Mandol					
0	0.22	0.16	0.00	0.00	2.41 ± 0.0004
10	0.17	0.13	0.00	0.00	2.29 ± 0.003
20	0.20	0.15	0.00	0.00	2.08 ± 0.0025
30	0.14	0.11	0.00	0.00	2.07 ± 0.0033
40	2.53	1.89	0.12	0.00	2.07 ± 0.0018
50	0.17	0.13	0.00	0.00	2.05 ± 0.0019
60	0.22	0.17	0.02	0.00	2.09 ± 0.0026
70	0.30	0.22	0.02	0.00	2.13 ± 0.0009
Ave.	0.49	0.37	0.20	0.00	2.15 ± 0.0000
Hajeon					
0	0.45	0.38	0.02	0.00	2.08 ± 0.0004
5	0.43	0.37	0.02	0.00	2.08 ± 0.0024
10	0.42	0.36	0.02	0.04	2.04 ± 0.003
15	0.55	0.48	0.03	0.02	2.00 ± 0.001
20	0.54	0.47	0.03	0.01	2.04 ± 0.0029
25	0.55	0.45	0.03	0.00	2.10 ± 0.0028
Ave.	0.49	0.42	0.03	0.01	2.06 ± 0.0000

**Fig. 5:**
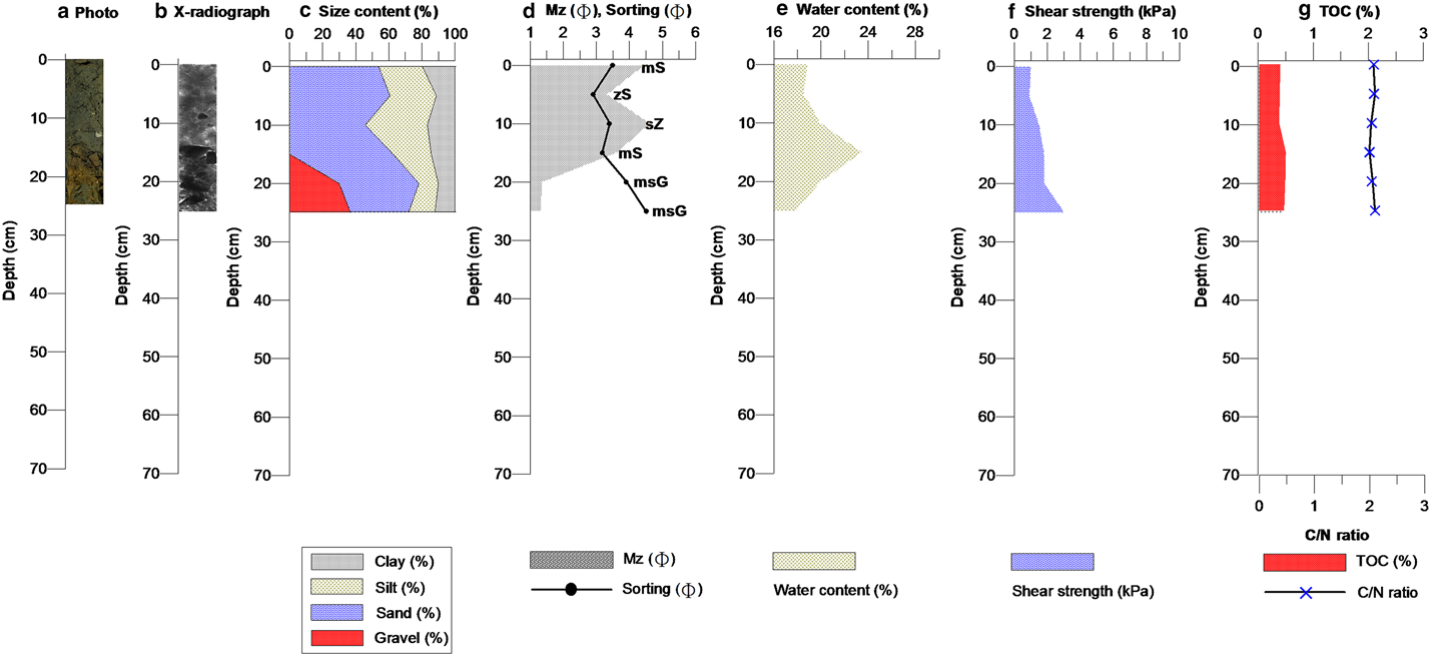
**a** Cross-sectional photographs showing, **b** a soft X-radiograph, **c** textural composition, **d** mean grain size and sorting, **e** water content, **f** shear strength, and **g** total organic carbon and C/N ratio in the sediment core from Hajeon

At Hajeon, the general color of the core was a dark olive gray (3Y/2) using soil color (Munsell 2000), which was darker than the Mandol core. Gravel was found in the lower layer at a depth of 20–25 cm. It was also detected on the X-radiograph (Fig. 5; Additional file 9). The core sample consisted of Mz 1.31–4.44 $\Phi$ (average 3.11 $\Phi$); sorting was 2.90–4.51 $\Phi$ (average 3.56 $\Phi$) with muddy sand, sandy silt, muddy sandy, and muddy sandy gravel, whereas its WC averaged 19.77 % and its shear strength averaged 1.7 kPa. The top part of the core was muddy sand. The TOC and TN varied with depth, but were generally about 0.42 % TOC and 0.002 g/cm^3^ or less TN (Tables 2, 3; Fig. 5).

When the proportions of sand, silt, and clay, disregarding gravel, among the Mandol and Hajeon samples were compared, it was found that Mandol was primarily composed of sand while the Hajeon sample was a mixture of sand. The organic carbon contents of sediments are related to the particle size, water depth, and sediment rates. Generally, the smaller the particles are, the larger the organic carbon retention capacity becomes. Given regular inflow to the bottom from the outer layer, the deeper the sediment is, the lower the organic carbon content would be assumed to become. The quicker sediments accumulate, the greater the retention of organic carbon can be. Generally, sediments are categorized according to particle size, mineral composition, and the concentration of carbonate and other organic matter contained within (Carter and Gregorich 2006; Udden 1914). The finer particles are, the higher the concentration of heavy metals and organic matter contained in the sediment, and likewise, the coarser they are, the lower the organic matter and heavy metal concentrations. Previous studies have used a C/N ratio of 4–10 to indicate a marine algae origin of organic matter and values >20 to indicate an origin from terrigenous vascular land plants. The distribution of C/N ratios in the cores indicated that the sediments originated from marine algae (Martinelli et al. 1999; Meyers 1994; Krishnamurthy et al. 1986; Müller 1977).

### In air sparging (IAS) experiment

**Fig. 6:**
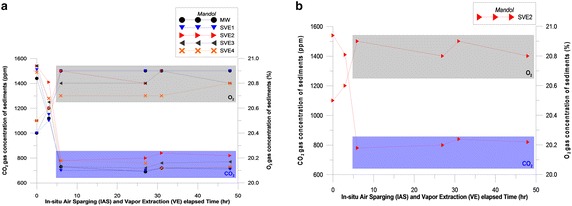
CO_2_ and O_2_ concentrations in Mandol. **a** MW, and SVE 1-4. **b** At the SVE2

During the 48 h experiment at the Mandol site, H_2_S and the LEL were not detected, but VOC was detected at a concentration of 0.1–2.0 ppm at the MW during the initial 30 min. At SVE wells 1–4, CO_2_ and O_2_ concentrations varied through the ranges of 690–1540 ppm and 20.4–20.9 %, respectively (Table 4; Fig. 6; Additional file 10).

**Table 4:** Temperature, radiation intensity, and humidity during the *In situ* air sparging (IAS) and Soil vapor extraction (SVE) experiments in Gomso Bay on the west coast of Korea

Content		Start date	Finish date
Mandol IAS + SVE	Date	31 May 2011 15:00	1 June 2011 15:00
	Temperature (monitoring well)	21.0–27.0 °C	21.0–26.0 °C
	Radiation intensity	350–380 × 100 Lux	100–110 × 100 Lux
	Humidity	48.1 % (25.5 °C)	93.3 % (16.8 °C)
Hajeon IAS + SVE	Date	2 June 2011 17:00	3 June 2011 17:00
	Temperature (monitoring well)	28.0–35.0 °C	22.0–26.0 °C
	Radiation intensity	900–920 × 100 Lux	225–230 × 100 Lux
	Humidity	93.4 % (22.0 °C)	75.0 % (22.0 °C)

**Fig. 7:**
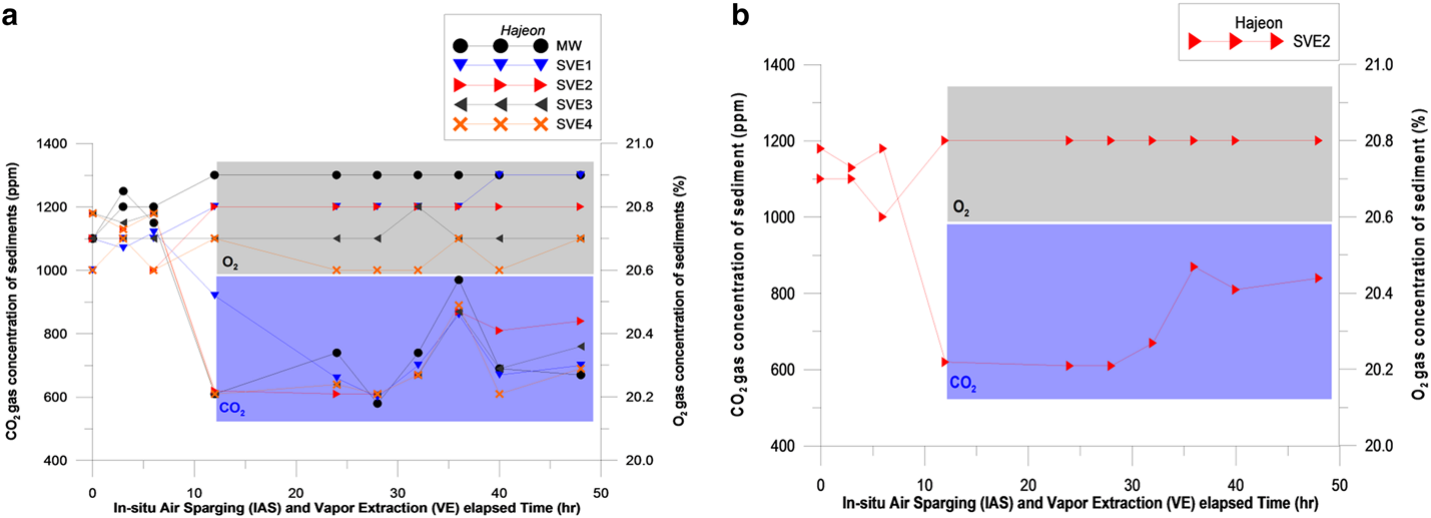
CO_2_ and O_2_ concentrations in Hajeon. **a** MW, and SVE 1–4. **b** At the SVE2

H_2_S and LEL were also not detected during the 48-h experiment at the Hajeon site, but VOC was detected at a concentration of 2.4–19.8 ppm at the MW from 12:00 to 19:00, in the middle of the 2 day experiment (local time). During the 48 h period at SVE wells 1–4, CO_2_ and O_2_ concentrations varied through the ranges of 580–1250 ppm and 20.6–20.9 %, respectively (Table 4; Fig. 7; Additional file 11).

Figure 6 shows the changes in CO_2_ and O_2_ concentrations during the 48-h experiment. IAS was used at the MW, 20 cm from SVE1, 40 cm from SVE2, 60 cm from SVE3, and 80 cm from SVE4. At each site, a rapid increase in the O_2_ concentration was detected, which stabilized over time. The reason for the stability in the increasing level of O_2_ at Mandol was due to the particles being relatively equally sized. Gravel, sand, and silt were mixed at Hajeon, and the particle sizes varied more than at Mandol. In addition, monitoring the concentrations of oxygen and carbon dioxide when injecting air into the MW produces important data in terms of evaluating whether the air-injection device delivers an adequate airflow to the desired sedimentary layer and that microbial metabolism proceeds appropriately. The reduction in CO_2_ and changes in the increase in O_2_ levels within 5 h of the start of the experiment (as shown in Figs. 6, 7) indicate that the injected air is conveyed well to the desired sedimentary layer.

The increase in oxygen levels indicates a direct response to IAS. Oxygen levels increased almost to the point of saturation 6.5 h after air injection began for IAS, near the MW. However, the initial rapid increase of oxygen near the sparging well may have been the result of short-circuiting, which could occur when air was injected into the well. The MW can work as a pressure sink resulting in short-circuiting. It can cause artificially high oxygen concentrations. Such short-circuiting of injected air has various geochemical influences on elements in an SVE well. Consequently, they should be measured immediately after the well cap is removed, or by using equipment installed in wells.

At Mandol, VOCs (0.1–2.0 ppm) were detected at the MW for 30 min after air was injected. At Hajeon, VOCs (2.4–19.8 ppm) were detected at the MW 12 h after air injection, and a concentration of 24.5 ± 2 ppm was detected 23 h after air injection. Pollutants were stripped from the aquifer and moved to the unsaturated area, sometimes causing VOC concentrations to increase. This means that organic pollutants within the aquifer were converted to a gaseous state and extracted from the site. Therefore, sediment treatment techniques such as SVE should be used together with IAS (Kim et al. 2000). The successful design and operation of AS and AS/SVE remediation systems is difficult due to the absence of reliable models that can accurately interpret the highly variable field conditions. The elevated concentrations of volatile materials in the initial stage may result from the effects of stripping by air injection. This effect is known to stabilize over time as shown by the later decrease in concentrations of VOCs. The experiments revealed that at Mandol, where particles are relatively equally sized, the concentration of VOCs decreased after the initial stage and did not increase again later. At Hajeon, the sediment particles were not regular and fine-sized, and therefore contained a large volume of organic material. The effect of tidal waters on the porosity of surrounding sediments also cannot be excluded when considering such an irregular phenomenon.

### Radius of influence (ROI)

An ROI refers to the maximum distance enabling the flow of air to be injected or extracted through injection or extraction wells. In the design stage, the ROI can be varied according to several factors comprising the physicochemical properties of soils, humidity, and times required for the remediation of corresponding sites. In this study, the ROI corresponded to the distance resulting from an incremental change of 0.1 inch H_2_O (2.5 mm H_2_O, 25 Pa) of air pressure measured from in situ experiments in an observation well equipped with a pressure gauge. The ROI at Mandol and Hajeon were calculated using Eq. (7). Generally, for an in situ experiment, the measurement of a pressure of 0.1 inch H_2_O (2.5 mm H_2_O, 25 Pa) is used to determine the ROI of the site. The ROI can be indirectly inferred from O_2_ changes in the AS operation zone (Al-maamari et al. 2011).

Measurement of the increase in dissolved oxygen (DO) levels should be conducted to determine the extent of the ROI in the MW by using field probes. Increases in DO levels due to the diffusion transport of oxygen will be noticeable during this study. In most cases, the DO levels increased because there was no air in the aquifer monitoring wells due to the overall change in the DO level flows. At the MW closest to the sparging well, the oxygen concentration was 20.9 %. This study assumed that an oxygen concentration of 20.6 % was the effective concentration for dictating the ROI.

7 $$ROI = \sqrt {\frac{Q \cdot (20.9{-}20.6\,\% )}{{\pi \cdot h \cdot k_{0} \cdot \theta_{a} }}} ,$$

*ROI* radius of Influence, *Q* air injection amount (m^3^/day), *h* aerotropic sediment depth (m), *k*_*0*_ coefficient of O_2_ (%/day), *θ*_*α*_, sediment volume rate.

At Mandol, the air injection (*Q*) was 28.8 m^3^/day, aerotropic sediment depth (*h*) was 0.5 m, the rate of O_2_ usage (*k*_*0*_) was 5.8 %/day, and sediment volume rate (porosity) (*θ*_*α*_) was 57.4 %. Similarly, at Hajeon, the air injection (*Q*) was 28.8 m^3^/day, aerotropic sediment depth (*h*) was 0.5 m, the rate of O_2_ usage (*k*_*0*_) was 14.0 %/day, and the sediment volume rate (porosity) (*θ*_*α*_) was 54.1 %. The ROI at Mandol and Hajeon were calculated to be 128.53 cm and 85.21 cm, respectively, according to Eqs. (8) and (9):

8 $$ROI,\, 128.53\, {\text{cm}}\,(in \,Mandol) = \sqrt {\frac{28.8 \cdot (20.9{-}20.6\,\% )}{\pi \cdot 0.5 \cdot 0.058 \cdot 0.574}} ,$$

9 $$ROI, \,85.21 \,{\text{cm}}\, (in\,Hajeon) = \sqrt {\frac{28.8 \cdot (20.9 {-} 20.6\,\% )}{\pi \cdot 0.5 \cdot 0.14 \cdot 0.541}} ,$$

Simulation of the primary material for accurate determination of the transfer coefficient was necessary for the first-order mass transfer coefficient. This model was developed to analyze and simulate decontamination by AS and AVE on the mass transfer process. The governing equations of mass transfer were solved numerically using a Galerkin’s finite element formulation (Rahbeh and Mohtar 2006).

**Fig. 8:**
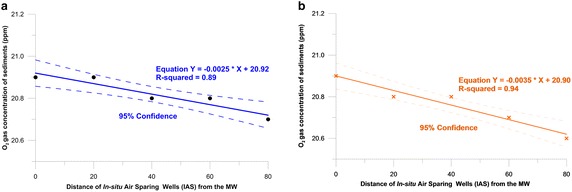
Estimated effective ZOI during IAS combined with SVE. **a** In Mandol. **b** In Hajeon

In Fig. 8 (Additional file 12), the O_2_ density of 20.6 % was selected as a determinant of the ROI (95 % confidence coefficient). Based on this, at Mandol and Hajeon, the ROI was calculated to be 128.0 cm (Equation, ${\text{Y}} = - 0.0025{\text{X}} + 20.92, R^{2} = 0.89$) and 85.7 cm (Equation, ${\text{Y}} = - 0.0035\,{\text{X}} + 20 . 9 0 ,\,{\text{R}}^{2} = 0.94$), respectively. The values determined from Eqs. (8) and (9) were 100.41 and 99.43 % (95 % confidence coefficient), respectively, which are very close. The reason for the ROI being small is likely to be the thin aquifer and thin screens at the test locations. At the MW closest to the sparging well, the oxygen concentration was 20.9 %, which was similar to that at the sparging well, but at distances farther away, the concentration tended to decline. The ROI after the insertion of air to sediments is a consequence of the depth, pressure, and flow rate of the air, selection of injection pulsing or continuous injection, and the distribution of sediment sizes. Therefore, the IAS method in pervasive sediments is likely to be more effective than in a channelized site such as Hajeon, which is a sandy area.

**Fig. 9:**
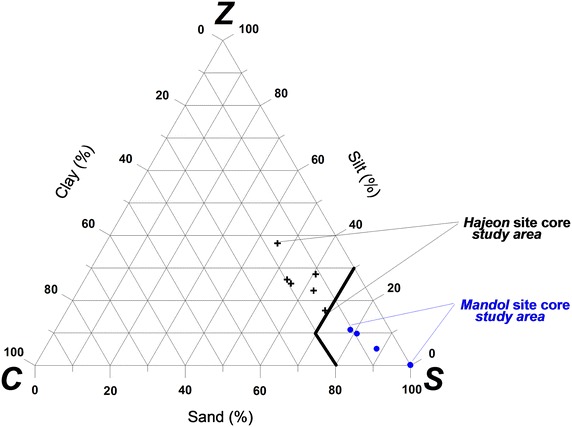
Distribution of sand, silt, and clay in cores taken from the tidal flat sediments

The ROIs of 128.53 and 85.21 cm for the sites of Mandol and Haejeon that were explored in this study approximated the minimum design ROI. The reasons behind such approximations can be inferred as follows. When the proportions of sand, silt, and clay (disregarding gravel) in the Mandol and Hajeon samples were compared, Mandol was primarily composed of sand, whereas Hajeon was a mixture of sand and silt (Fig. 9; Additional file 13; Folk and Ward 1957). Increased air volume in sand sediments is directly related to increased permeability. Several studies presented evaporation (remediation) characteristics from soils such as silt and clays, which exhibit higher porosities but have pore size distributions skewed towards smaller pores, show lower evaporation (remediation) rates than sand. Of these, sediments aggregation and porosity were the most important (Fine and Yaron 1993).

Within ROI, oxygen can potentially be transferred to resident microorganisms participating in biological degradation processes (Surendhiran et al. 2015; Chikere et al. 2011; US Army corps of Engineers 2002). The radius of oxygen transference is the maximum distance of the transference of oxygen in air injected through injection wells for biological degradation of contaminants. An ROI is expressed as a circle centered around the injection or extraction wells and thus, the radius of this circle denotes the ROI (EPA 2004). Therefore, the Mandol site (ROI of 128.53 cm) was concluded to have better oxygen transference conditions for biological degradation than the Haejeon site (ROI of 85.21 cm).

The ROI estimated through in situ experiments can be exploited as an important determinant in the design of IAS and SVE systems. Ways to increase the ROI of the Haejeon site (85.21 cm) can be found in existing experimental methods (Edwards and Jones 1994). In an experiment conducted to increase the ROI through a pneumatic scheme, a top impervious layer of 10 cm depth was prepared on a mixture of bentonite and soils in an unsaturated zone of 3 m depth. Thus, additional treatment employing pneumatic schemes can increase the extent of ROI at the Hajeon site. However, since there can be differences between the generally known design ROI and the ROI measured in this study in terms of the following factors, empirical judgment is also important. Before an IAS system can be utilized, sediment characterization of the site should be conducted. In addition, a sparging well should always be established to determine the appropriate sparge air-injection pressure, screen depth, flow rate, and the effective ROI. The VOC, O_2_, and CO_2_ concentrations in the SVE wells provided the best indication of the air sparge effectiveness and the air sparge ROI. However, subsurface conditions vary between sites, and all of the pertinent parameters should be measured during a pilot-scale experiment. In addition, these parameters should be measured periodically during the operation of an IAS system.

Besides the factors measured and analyzed in this study, there may be unidentified factors such as the following. With respect to the time and/or cost, the unidentified factors can be summarized as follows: (1) contaminant type, (2) operation of sparge pulsing mode, (3) number of wells, (4) maximum biodecay rate, and (5) aquifer organic carbon content. In addition, for each field site, (6) the dwell time of sparged air, (7) contaminant equilibrium influencing experiments, (8) contaminant phase distribution, (9) oxygen availability to microbes and (10) pulsed air injection or continuous airflow (Michael et al. 2002).

## Conclusions

The Mandol core sample consisted of 1.60–3.41 $\Phi$ (average 2.30 $\Phi$) sand and muddy sand; its WC was on average 15.10 % and its shear strength was on average 1.1 kPa. The Hajeon core sample consisted of 1.31–4.44 $\Phi$ (average 3.11 $\Phi$) muddy sand, sandy silt, muddy sandy, and muddy sandy gravel; its WC was on average 19.77 % and its shear strength was on average 1.7 kPa. The Mandol sample was close to sand, while Hajeon was a mixture of sand and silt.

This study assumed an oxygen concentration of 20.6 % to be the effective concentration for the experiment. Based on this O_2_ content, the effective ROI at Mandol was estimated to be 128.0 cm, and at Hajeon, it was estimated to be 85.7 cm. The smaller effective ROI was likely to be caused by a thin aquifer and the thin screens of the sparing well. At the MW closest to the sparging well, the oxygen concentration was 20.9 %, which was similar to that at the sparging well, and this concentration decreased with distance from the source, i.e., the distance and dissolved oxygen were inversely related. At Mandol, VOCs (0.1–2.0 ppm) were detected at the MW for 30 min after air was injected. At Hajeon, VOCs (2.4–19.8 ppm) were detected at the MW for 12 h after air was injected, and a concentration of 24.5 ± 2 ppm was detected 23 h after air injection. Pollutants were stripped from the saturated zone and volatilized to the unsaturated area, sometimes causing VOC concentrations within the sediments to increase.

## Additional files

10.1186/s40064-016-3026-3 IAS experimental sites in Gomso Bay.
10.1186/s40064-016-3026-3 Textural composition of core sediments from Mandol and Hajeon.
10.1186/s40064-016-3026-3 Organic character of core sediments from Mandol and Hajeon.
10.1186/s40064-016-3026-3 Weather data during the IAS and SVE.
10.1186/s40064-016-3026-3 Schematic diagram of the monitoring, IAS and SVE.
10.1186/s40064-016-3026-3 Location of IAS experiments combined with SVE.
10.1186/s40064-016-3026-3 Photograph of an IAS experiment combined with SVE.
10.1186/s40064-016-3026-3 Cross-sectional showing in the sediment core from Mandol.
10.1186/s40064-016-3026-3 Cross-sectional showing in the sediment core from Hajeon.
10.1186/s40064-016-3026-3 CO_2_ and O_2_ concentrations in Mandol.
10.1186/s40064-016-3026-3 CO_2 _and O_2 _concentrations in Hajeon.
10.1186/s40064-016-3026-3 Estimated effective ZOI during IAS combined with SVE.
10.1186/s40064-016-3026-3 Distribution of sand, silt, and clay from the tidal flat sediments.

## Abbreviations

AS: air sparging
BD: bulk density
DO: dissolved oxygen
IAS: in situ air sparging
LEL: lower explosion level
MHHW: mean higher high water
MW: monitoring well
MC: monte carlo
NIST: National Institute of Standards and Technology
ROI: radius of influence
ROI_SAT_: radius of influence in the saturated zone
ROI_VAD_: radius of influence in the vadose zone
S: sand
Z: sand silt
sZ: sandy silt
zS: silty sand
SRM: soil reference material
SVE: soil vapor extraction
TC: total carbon
TN: total nitrogen
TOC: total organic carbon
VOCs: volatile organic compounds
WC: water content
